# Altered brain activity associated with premature ejaculation improved by electroacupuncture in rats

**DOI:** 10.1093/sexmed/qfae047

**Published:** 2024-08-31

**Authors:** Ning Wu, Jian-huai Chen, Tong Wang, Bai-bing Yang, Si-yan Xing, Song-zhan Gao, Da-wei Ni, Guang-jun Du, Tao Song, You-feng Han, Guo-hai Sun, Qing-qiang Gao, Chun-lu Xu, Yu-tian Dai

**Affiliations:** Department of Andrology, Nanjing Drum Tower Hospital Clinical College of Nanjing University of Chinese Medicine, Nanjing 210008, China; Department of Urology, The People’s Hospital of Jiaozuo, No. 263 Jiefang Middle Road, Jiaozuo 454002, China; Department of Andrology, Jiangsu Province Hospital of Chinese Medicine, Affiliated Hospital of Nanjing University of Chinese Medicine, Nanjing 210029, China; Department of Andrology, Nanjing Drum Tower Hospital Clinical College of Nanjing University of Chinese Medicine, Nanjing 210008, China; Department of Andrology, Nanjing Drum Tower Hospital, The Affiliated Hospital of Nanjing University Medical School, Nanjing 210008, China; Department of Andrology, Nanjing Drum Tower Hospital, The Affiliated Hospital of Nanjing University Medical School, Nanjing 210008, China; Department of Andrology, The Third Affiliated Hospital of Zhengzhou University, Zhengzhou 450052, China; Department of Andrology, Nanjing Drum Tower Hospital Clinical College of Nanjing University of Chinese Medicine, Nanjing 210008, China; Department of Andrology, Nanjing Drum Tower Hospital, The Affiliated Hospital of Nanjing University Medical School, Nanjing 210008, China; Department of Andrology, Nanjing Drum Tower Hospital, The Affiliated Hospital of Nanjing University Medical School, Nanjing 210008, China; Department of Andrology, Nanjing Drum Tower Hospital, The Affiliated Hospital of Nanjing University Medical School, Nanjing 210008, China; Department of Andrology, Nanjing Drum Tower Hospital, The Affiliated Hospital of Nanjing University Medical School, Nanjing 210008, China; Department of Andrology, Nanjing Drum Tower Hospital, The Affiliated Hospital of Nanjing University Medical School, Nanjing 210008, China; Department of Andrology, Nanjing Drum Tower Hospital, The Affiliated Hospital of Nanjing University Medical School, Nanjing 210008, China; Department of Andrology, Nanjing Drum Tower Hospital Clinical College of Nanjing University of Chinese Medicine, Nanjing 210008, China

**Keywords:** electroacupuncture, fractional amplitude of low frequency fluctuation, functional magnetic resonance imaging, premature ejaculation, regional homogeneity, resting-state

## Abstract

**Background:**

Premature ejaculation (PE) is linked with abnormal brain activity that is modifiable by electroacupuncture (EA).

**Aim:**

In this study we aimed to explore the central pathological mechanism underlying EA in treating PE.

**Methods:**

Six-week-old male Sprague–Dawley rats were divided into a PE group (n = 8) and a control group (n = 8) according to ejaculatory frequency during copulatory behavior. All rats underwent EA at the Zusanli acupoint (ST-36) for 4 weeks. Magnetic resonance imaging data were collected before and after EA.

**Outcomes:**

The behavioral parameters, plasma norepinephrine levels, fractional amplitude of low frequency fluctuation (fALFF), and regional homogeneity (ReHo) were evaluated.

**Results:**

The PE group ejaculated more times with shorter latency compared with controls. After EA, the ejaculation frequency of the PE group decreased, and the ejaculation latency period increased, with no changes observed in the control group. Norepinephrine levels were higher in the PE group than in the controls and were positively correlated with ejaculation frequency and negatively correlated with ejaculation latency. The PE group showed lower fALFF in the right striatum and higher ReHo in the brainstem compared with controls. After EA, controls showed decreased fALFF in the right striatum, left olfactory bulb, and dorsal fornix and increased ReHo in the right interpeduncular nucleus, as well as decreased ReHo in the left striatum, prelimbic system, right basal forebrain region, septal region, and olfactory bulb, while the model group exhibited increased fALFF in the right hypothalamic region, decreased fALFF in the left globus pallidum and right basal forebrain region and increased ReHo in the right interpeduncular nucleus, as well as decreased ReHo in the left striatum, olfactory bulb, basal forebrain region, dentate gyrus, right dysgranular insular cortex, and striatum. Compared with the controls after EA, the model group showed increased ReHo of the right hypothalamic region and decreased ReHo of the right dysgranular insular cortex.

**Clinical Implications:**

These findings might enhance the understanding of PE and contribute to new, targeted therapies for PE.

**Strengths and Limitations:**

The therapeutic effects might be achieved by EA inhibiting the activity in brain regions involved in ejaculatory behavior. However, the curative effect of acupuncture might be underestimated due to some curative effects of sham acupuncture used in the control group.

**Conclusion:**

In conclusion, the ejaculatory frequency of rats may be reduced and ejaculation latency could be extended by EA at ST-36, which might be achieved by the effects of this treatment on brain activity.

## Introduction

Premature ejaculation (PE) is a common male sexual disorder characterized by short ejaculatory latency that affects the quality of life for 20%-30% of men worldwide [1, 2]. Recent studies have shown that PE is related to abnormal activities of the central and peripheral neural systems [3–5]. Therefore, local anesthetics and selective serotonin reuptake inhibitors (SSRIs) have been shown to effectively treat PE by prolonging the intravaginal ejaculation latency time (IELT) and improving the sexual satisfaction of patients [6, 7]. However, after treatment with local anesthetics, 57% to 74% of PE patients achieved an improvement in IELT (had more than 2 minutes of IELT) [8, 9], while only 47% to 51% of patients had IELT of ≥2 minutes after receiving dapoxetine treatment [10]. In our previous studies, PE patients were classified into four subtypes based on their neuroelectrophysiological characteristics: peripheral type, sympathetic type (central type), mixed type, and other types [11]. Among these patients, peripheral, sympathetic, and mixed PE patients had an effective rate of over 80% when they were treated with local anesthetics and/or dapoxetine; however, the effective rate of patients with other types of PE was less than 50% [11]. Therefore, in-depth research on the pathogenesis and treatment methods of PE is urgently needed.

Many studies have shown that PE is associated with abnormalities of the sympathetic nervous system [12–14]. The hypothalamic paraventricular nucleus is an important region that regulates the activity of the sympathetic nervous system and is also involved in control of the ejaculatory reflex [15, 16]. In our previous animal studies, the ejaculatory frequency of rats was improved by inhibiting the sympathetic nervous activity, which was achieved by inhibiting the aspartate receptor in the paraventricular nucleus or activating the hypothalamus γ-aminobutyric acid receptors [17, 18]. Therefore, how to inhibit the central sympathetic nervous system is a key issue in the treatment of PE. Acupuncture is a nonpharmacological therapy to prevent and treat diseases by using needles to stimulate acupoints with different manipulation techniques under the guidance of the theory of Chinese Medicine [19]. This treatment method is widely recognized internationally and has been used in the treatment of various diseases, including PE [20, 21]. Acupuncture is a therapy that uses needles to stimulate acupoints by lifting and twisting stimulation. The main acupuncture points used for treating PE are ST-36 Zusanli, BL-23 Shenshu, LR-3 Taichong, and SP-6 Sanyinjiao [22]. Zusanli, an acupoint at stomach 36 (ST-36), belongs to the acupoints of the stomach meridian and is often used to treat diseases of the Qi deficiency type. In particular, a recent study has shown that electroacupuncture at ST-36 can stimulate the vagus nerve, reducing sympathetic nerve excitability and thereby improving gastric motility [23]. In addition, acupuncture at ST-36 may effectively treat hypertension by inhibiting the activity of sympathetic nerves and enhancing parasympathetic nerves [24]. Previous studies have revealed that ejaculation latency (EL) could be related to the basic state of the sympathetic nervous system during sexual behavior [12, 13, 25]. However, the underlying cerebral central mechanism is unclear.

Resting-state functional magnetic resonance imaging (rs-fMRI) is a noninvasive neuroimaging method that has been used to explore the mechanisms of action underlying acupuncture [26, 27]. Changes of regional brain activity related to acupuncture stimulation have been identified in previous studies [28, 29]. Acupuncture showed influences on the activity of brain areas, including the somatosensory cortices, limbic system, basal ganglia, brain stem, and cerebellum [29]. In addition, acupuncture stimulation may adjust the functional connectivity of brain networks, including the limbic–paralimbic–neocortical network, brainstem, cerebellum, subcortical region, and hippocampus [26]. Therefore, we hypothesized that short ejaculatory latency might be associated with altered brain activity in rats, which could be improved by EA. In this study, rs-fMRI data were collected from rats with short ejaculatory latency and those with normal ejaculatory latency. Measurements of the fractional amplitude of low frequency fluctuation (fALFF) and regional homogeneity (ReHo) were calculated and compared to explore the central pathological mechanism underlying EA in the treatment of PE.

## Materials and methods

### Animals

The ethics of the animal experimentation was approved by Nanjing Drum Tower Hospital (No. DWSY-22149276). Six-week-old Sprague–Dawley rats weighing 200-250 g were provided by the Experimental Animal Center of Nanjing Drum Tower Hospital. To adapt to the environment, rats arrived at the laboratory at least 2 weeks before the start of the experiment. Rats were raised in a 12-hour light/dark cycle environment with a temperature range of 18°C-22°C and relative humidity of 40%-50%. In addition, rats were kept in separate cages, with free access to food and water.

### Establishment of a PE model

Bilateral ovariectomy was performed in female rats after they had been anesthetized with isoflurane (0.5-1.5%). One week later, artificially induced estrus was established in female rats with the following steps: (1) 0.1 mL sesame oil and 20 μg estradiol benzoate were injected subcutaneously 48 hours before a mating test and (2) 0.1 mL sesame oil and 500 μg progesterone were injected subcutaneously 4 hours before a mating test.

Ejaculatory behavior was observed according to the following steps. Mating tests were performed in a 60 × 40 × 30–cm^3^ cage from 20:00 to 21:00. After the rats adapted to the environment for 5 minutes, the mating processes of male rats with estrus female rats were observed within 30 minutes. The following parameters were recorded: mount latency (ML; time of first riding behavior), insertion latency (IL; time of first insertion behavior), EL (time of first ejaculatory behavior), mount frequency (MF), insertion frequency (IF), ejaculation frequency (EF), post-ejaculatory interval (PEI), and intromission ratio (IR). According to previous studies, the EF of rats tends to stabilize after the third mating. Therefore, 3 mating tests were conducted on each male rat. Male rats with 1 < EF < 3 times in the 3 tests were selected as the control group while those with EF ≥ 3 times in the 3 tests were classified as the model group.

### Electroacupuncture protocols

After the male rats were anesthetized with isoflurane (0.5%-1.5%), stainless steel needles (0.30-mm diameter, 25-mm length) were inserted perpendicularly into the muscle layer at the ST-36 acupoint, which is located in the anterolateral portion of the hindlimb, in the middle of the cranial tibial muscle, 5 mm below the capitulum fibulae and 3-4 mm deep from the skin surface, and is innervated by the deep fibular nerve. The anode and cathode of a biological multichannel stimulator (Hwata SDZ-II B, Suzhou, China) were connected to the needles on both sides (continuous wave: 2 Hz, ≤2 mA, 30-minute duration) which elicited a slight muscle contraction or movement of the paw, with daily treatment for a total of 4 weeks. All procedures were performed by the same researcher.

### Plasma norepinephrine measurement

Trunk blood was harvested from the postcava and centrifuged at 2000*g* for 10 minutes at 4°C. The plasma was harvested and stored at −80°C for subsequent use. Plasma norepinephrine (NE) concentrations were measured using an enzyme-linked immunosorbent assay kit (catalog No. E0907Ge, Nanjing Drum Tower Hospital Andrology Laboratory, Nanjing, China). The experimental procedures for enzyme-linked immunosorbent assay were performed in accordance with the manufacturer’s manuals.

### MRI data acquisition

The MRI data were acquired with a 9.4 T MRI scanner (Bruker BioSpec 94/20 USR, Ettlingen, Germany). The rs-MRI data were acquired with the following parameters: repetition time (TR) = 2000 milliseconds; echo time (TE) = 7.2 milliseconds; slice thickness = 0.6 mm; slice gap = 0.6 mm; field of view (FOV) = 45 × 45 mm^2^; matrix = 100 × 100; flip angle (FA) = 30°; number of slices = 58; volume = 240. In addition, T2 images were acquired with the following parameters: TR = 8120 ms; TE = 38 milliseconds; slice thickness = 0.5 mm; slice gap = 0.5 mm; FOV = 350 × 350 mm^2^; matrix = 100 × 100; FA = 180°; number of slices = 68.

### MRI data preprocessing

The MRI data preprocessing was performed with Statistical Parametric Mapping (SPM8) and Data Processing Assistant for Resting-State fMRI (DPARSF) software [30] based on MATLAB, with the following steps: (1) data format conversion; (2) discarding the first 10 functional time points; (3) slice timing correction; (4) head motion correction; (5) functional and structural image reorientation; (6) structural image coregistration to corresponding functional images; (7) structural images segmented into grey matter, white matter, and cerebrospinal fluid; (8) functional images spatially normalized into the standard space.

### Calculations of fALFF and ReHo

The steps of fALFF calculation were as follows: (1) spatial smoothing; (2) linear trend; (3) regressed out nuisance signals (head motion parameters); (4) converting time series to the frequency domain with the fast Fourier transform algorithm; (5) calculating square roots of the power spectrum and averaging the square roots of all voxels, regarded as ALFF; (6) calculating the ratios of the power spectrum at the low-frequency range to that of the entire frequency range, regarded as fALFF. In addition, to improve the normality distribution, fALFF values were standardized into z-standardized fALFF values by use of Fisher’s r-to-z transformation. The measure of fALFF reflects the magnitude of spontaneous neural activity by measuring changes in the BOLD (blood oxygen level–-dependent) signal.

The steps of ReHo calculation were as follows: (1) linear trend; (2) temporal band-pass filtering; (3) regressed out nuisance signals (head motion parameters); (4) calculated synchronization of the time series of a given voxel with those of its 26 adjacent neighbors (Kendall’s coefficient of concordance, also called ReHo). In addition, to improve the normality distribution, ReHo values were standardized into zReHo values by Fisher’s r-to-z transformation. The ReHo method was used to measure the functional synchronization of a voxel with its close neighbors during the resting state.

### Statistical analysis

Differences of behavioral parameters were compared between groups by use of two-sample *t*-tests (between the model and control groups) and paired *t*-tests (between the model/control group before and after treatment) based on the software of Statistical Package for the Social Sciences (SPSS). *P* < .05 was considered to indicate statistically significant differences. In addition, the software of SPM was used to compare the differences of fALFF and ReHo between groups by two-sample *t*-tests (between the model and control groups) and paired *t*-tests (between the model/control group before and after treatment). The significant differences were set at *P* < .005 and the minimum cluster size was 5 voxels, uncorrected.

## Results

### Comparison of behavioral parameters between groups

Of the 80 male rats, 21 were removed from the study due to the absence of mating behavior in six mating trials. Based on the last 3 sexual behavior tests, 8 rats with EF ≥ 3 times were classified into the model group, while there were 18 rats with 1 < EF < 3 times and we randomly selected 8 as the control group. Before treatment, higher ML, EF, and IR, as well as lower IL, EL, MF, and PEI, were found in the PE group than in the control group. After treatment, the PE group showed decreased ML and EF and increased MF and also exhibited lower IL, EL, and MF compared with the control group. However, there were no significant changes in EF and EL in the control group before and after treatment (Table 1).

**Table 1:** Comparison of behavioral parameters and plasma NE levels between groups.

	**Controls**		**PE treated**	
	**Before treatment (n = 8)**	**After treatment (n = 8)**	**Before treatment (n = 8)**	**After treatment (n = 8)**
ML, s^a,b^	6.08 ± 4.00	8.71 ± 5.78	18.92 ± 7.22	8.88 ± 3.51
IL, s^a,c^	39.13 ± 14.16	39.88 ± 14.95	24.29 ± 9.45	20.96 ± 9.59
EL, s^a,b,c^	626.04 ± 89.60	635.92 ± 84.19	262.54 ± 48.03	558.29 ± 71.28
MF^a,b,c^	21.96 ± 8.40	22.29 ± 5.505	7.96 ± 4.05	12.83 ± 4.29
IF	7.42 ± 3.78	7.33 ± 2.68	6.96 ± 3.29	6.92 ± 2.96
EF^a,b^	1.96 ± 0.86	1.79 ± 0.72	3.83 ± 1.05	2.04 ± 0.91
PEI, s^a^	509.75 ± 72.10	444.96 ± 84.97	489.58 ± 68.02	508.79 ± 70.8
IR, %^a^	0.25 ± 0.06	0.48 ± 0.15	0.35 ± 0.10	0.26 ± 0.10
NE, nmol/L^a,b,c^	7.33 ± 0.16	7.18 ± 0.22	9.12 ± 0.29	8.03 ± 0.23

Abbreviations: EF, ejaculation frequency; EL, ejaculation latency (time of first ejaculatory behavior); IF, insertion frequency; IL, insertion latency (time of first insertion behavior); IR, intromission ratio; MF, mount frequency; ML, mount latency (time of first riding behavior); NE, norepinephrine; PE, premature ejaculation; PEI, postejaculatory interval;

^a^Significant differences between PE and controls before treatment.

^b^Significant differences between PE before and after treatment.

^c^Significant differences between PE and controls after treatment. *P* values were obtained with two-sample *t*-tests. The significant level was set at *P* < .05.

### Correlation between NE and copulatory behavior parameters

**Figure 1:**
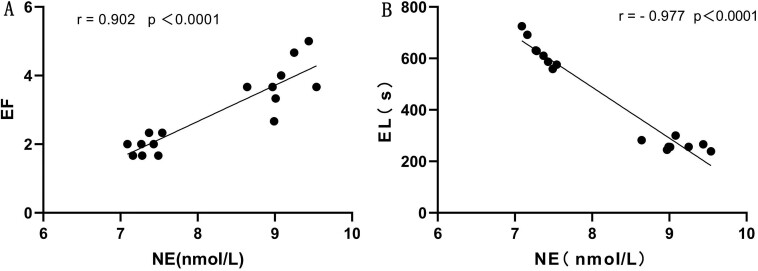
Correlation of the NE level with ejaculation frequency (A) and ejaculation latency (B). The plasma NE level was used as the indirect index of sympathetic neural activity. Both EL and EF were correlated with plasma NE (both *P* < .01). EF, ejaculation frequency; EL, ejaculation latency; NE, norepinephrine (nmol/L).

Before treatment the plasma NE levels were recorded as 9.12 ± 0.29 nmol/L in the PE group and 7.33 ± 0.16 nmol/L in the control groups and after treatment were 8.03 ± 0.23 nmol in the PE group and 7.18 ± 0.22 nmol/L in the controls (Table 1). The NE level in the PE group was significantly higher than that in control group (*P* < .05), and after treatment, the level in the PE group was significantly decreased (*P* < .05), whereas there was no significant change in control group. Moreover, as displayed in Fig. 1, the plasma NE levels were positively correlated with EF (*r* = 0.90, *P* < .01), but negatively correlated with EL (*r* = −0.98, *P* < .01).

### Brain regions showed altered regional activity

####  

##### PE group vs control group before treatment

**Figure 2:**
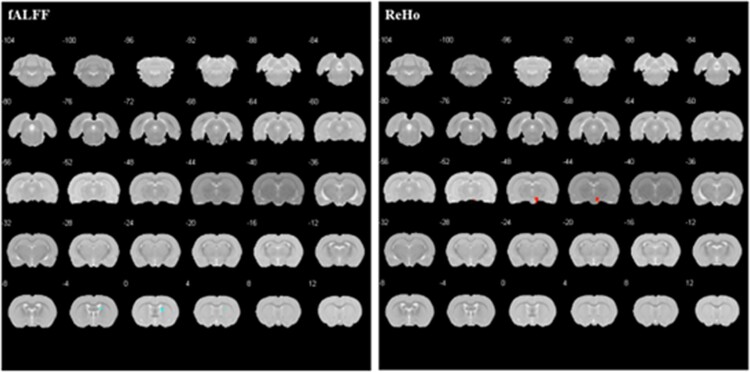
Altered brain regions in PE rats compared with controls before treatment. The significant differences were set at *P* < .005 and the minimum cluster size was 5 voxels, uncorrected. fALFF, fractional amplitude of low frequency fluctuation; PE, premature ejaculation; ReHo, regional homogeneity.

Compared with the control group before EA, decreased fALFF of the right striatum and increased ReHo of the right brainstem were found in the model group before EA (Table 2; Fig. 2).

**Table 2:** Brain regions showing altered regional activity.^a^

**Brain regions**	**Peak MNI coordinates**			**Clusters**	**Peak *t* values**
	***x***	***y***	***z***		
PE vs controls before treatment					
fALFF					
Striatum R	20	0	24	6	−4.11
ReHo					
Brainstem R	12	−44	−12	12	3.80
Controls after vs before treatment					
fALFF					
Striatum R	24	24	6	5	−3.65
Olfactory bulb L	−4	52	12	7	−4.34
Dorsal fornix L	−8	−20	24	6	−4.05
ReHo					
Interpeduncular nucleus R	4	−68	−30	6	4.05
Striatum L	−36	12	30	6	−3.85
Prelimbic system L	−16	44	30	16	−4.51
Basal forebrain region R	16	8	−24	5	−3.84
Septal region R	16	0	24	14	−3.73
Olfactory bulb R^1^	4	56	24	11	−3.89
Olfactory bulb R^2^	16	40	12	10	−3.53
PE after vs before treatment					
fALFF					
Hypothalamic region R	8	−24	−18	5	3.48
Globus pallidum L	−20	−4	12	5	−4.54
Basal forebrain region R	4	8	0	7	−4.77
ReHo					
Interpeduncular nucleus R	12	−60	−18	6	4.88
Striatum L	−16	4	24	15	−4.02
Olfactory bulb L	−20	52	24	25	−5.17
Basal forebrain region L	−24	0	−6	23	−3.48
Dentate gyrus L^1^	−44	−56	−6	9	−4.40
Dentate gyrus L^2^	−8	−32	30	5	−3.78
Dysgranular insular cortex R	56	0	6	11	−5.23
Striatum R^1^	16	8	18	9	−4.32
Striatum R^2^	36	8	30	5	−3.22
PE vs controls after treatment					
fALFF					
No significant differences					
ReHo					
Hypothalamic region R	8	−24	−18	9	4.63
Agranular insular cortex R	36	28	18	7	−4.07

Abbreviations: fALFF: fractional amplitude of low frequency fluctuation; L, left; MNI, Montreal Neurological Institute; PE, premature ejaculation; R, right; ReHo: regional homogeneity; *x*, *y* and *z*, coordinates of peak voxels of clusters in MNI space.

^a^Significant differences were set at *P* < .005 and the minimum cluster size was 5 voxels, uncorrected.

#### Controls after vs before treatment

**Figure 3:**
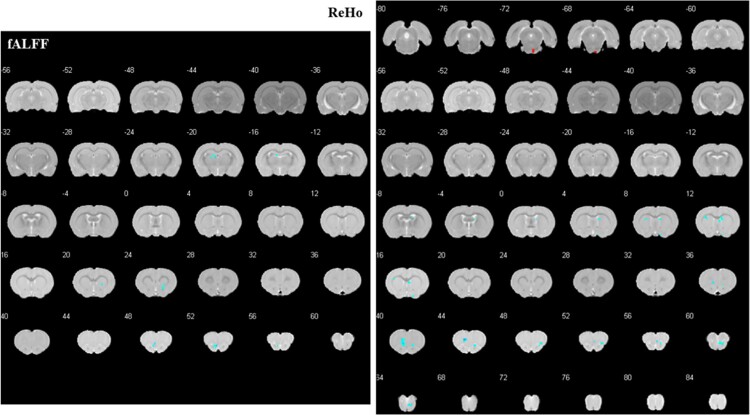
Altered brain regions in control rats after treatment compared with those before treatment. The significant differences were set at *P* < .005 and the minimum cluster size was 5 voxels, uncorrected. fALFF: fractional amplitude of low frequency fluctuation; ReHo: regional homogeneity.

After EA, the control group showed decreased fALFF in the right striatum, left olfactory bulb, and dorsal fornix and increased ReHo in the right interpeduncular nucleus, as well as decreased ReHo in the left striatum, prelimbic system, right basal forebrain region, septal region, and olfactory bulb. (Table 2; Fig. 3).

##### PE after vs before treatment

**Figure 4:**
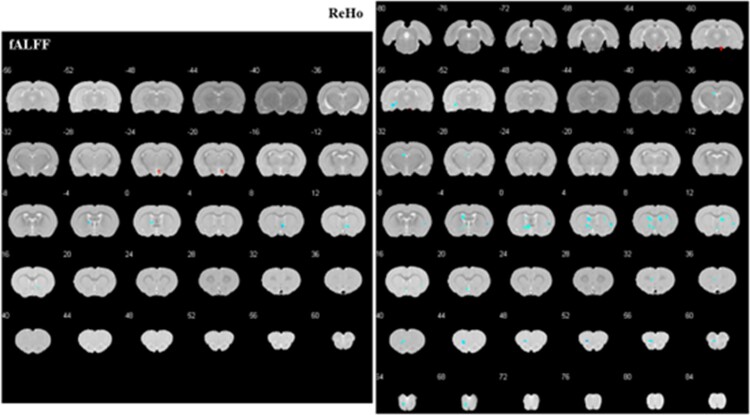
Altered brain regions in PE rats after treatment compared with those before treatment. The significant differences were set at *P* < .005 and the minimum cluster size was 5 voxels, uncorrected. fALFF, fractional amplitude of low frequency fluctuation; PE, premature ejaculation; ReHo, regional homogeneity.

After EA, the treatment group exhibited increased fALFF in the right hypothalamic region, decreased fALFF in the left globus pallidum and right basal forebrain region, and increased ReHo in the right interpeduncular nucleus, as well as decreased ReHo in the left striatum, olfactory bulb, basal forebrain region, dentate gyrus, right dysgranular insular cortex, and striatum. (Table 2; Fig. 4).

##### PE vs controls after treatment

**Figure 5:**
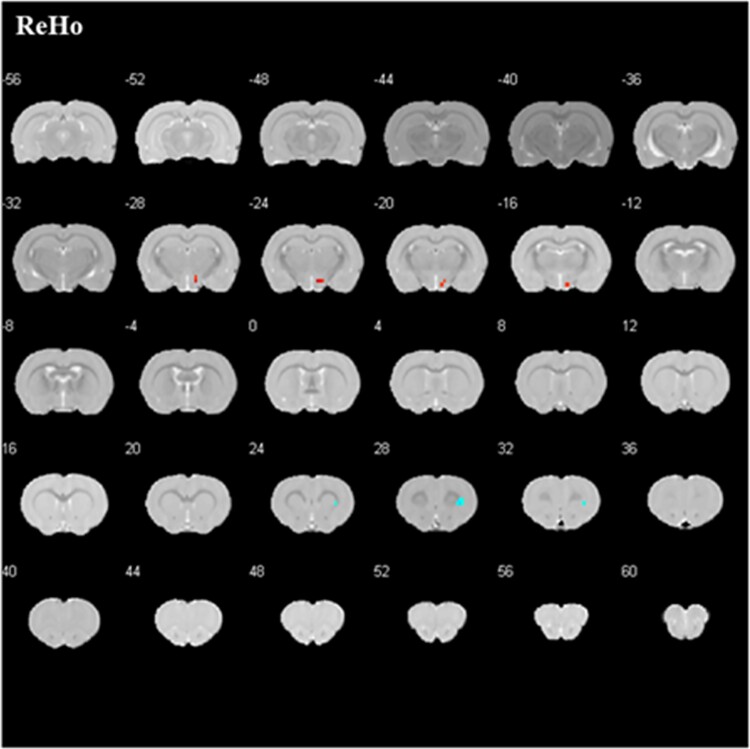
Altered brain regions in PE rats when compared with controls after treatment. The significant differences were set at *P* < .005 and the minimum cluster size was 5 voxels, uncorrected. fALFF, fractional amplitude of low frequency fluctuation; PE, premature ejaculation; ReHo, regional homogeneity.

Compared with the control group after EA, increased ReHo of the right hypothalamic region and decreased ReHo of the right dysgranular insular cortex were found in the treatment group after EA. (Table 2; Fig. 5).

## Discussion

To the best of our knowledge, this is the first reported study exploring the central pathological mechanism underlying EA at ST-36 in the treatment of PE rats. Altered intensity and concordance of regional brain activity were found in rats with higher ejaculation frequency, and these changes were modulated by EA at ST-36. Decreased brain activity was identified in both the treatment and control groups after EA, which suggested that brain activity could be inhibited by the treatment of EA at ST-36. All these findings provided new insights into understanding the pathological mechanism underlying PE and the mechanism underlying the effect of EA in treating PE at ST-36.

In this study, ejaculation latency was increased in rats after 4 weeks of EA treatment at ST-36. Acupuncture needles inserted into the body can produce the sensation of pain, which has therapeutic effects for many diseases [31]. Acupuncture and EA have been widely used to treat PE and can extend ejaculation latency by regulating the balance of qi and blood [32, 33]. In addition, the 5-hydroxytryptamine level and nerve sensitivity, which were associated with the occurrence of PE, could be regulated by EA at the acupoints Zusanli, Tianshu, and Taichong [34]. Previous study has shown that acupuncture has significant effects on improving the feeling of control over ejaculation and relieving distress in PE patients [22]. Considering the side effects of SSRIs, acupuncture may be noninferior to SSRIs, and the beneficial effects of acupuncture, including prolonging IELT and improving PEDT scores, have been identified in a previous study [22]. In addition, both previous human and animal studies have demonstrated that acupuncture can induce neuromodulating inputs into the brain [35, 36]. However, the changes of brain activity associated with acupuncture were unclear in both humans and animals with PE.

Brain regions showing responses to acupuncture, which included the prefrontal and cingulate cortex, amygdala, hippocampus, hypothalamus, insula, basal ganglia, and cerebellum, were detected in previous neuroimaging studies [26, 27]. The ST-36 acupuncture pointis located 3 cm below the knee joint on the anterior portion of the leg [37, 38]. Based on its functional characteristics, acupuncture at ST-36 is effective in the treatment of cognition, pain, motor, memory, learning, and other aspects [38]. A previous meta-analysis study showed that the bilateral rolandic operculum and cerebellum, as well as the right supramarginal gyrus, could be activated by acupuncture at ST-36, which might be related to action and perception [38]. In addition, acupuncture at ST-36 positively activated the left median cingulate gyrus and superior temporal gyrus, as well as the right inferior frontal gyrus (opercular part) of healthy subjects, which were related to pain, somatosensory functions, language, and emotion [37]. However, the neurobiologic substrates and mechanisms underlying the effects of acupuncture in the treatment of PE are unclear.

In this study, decreased intensity and concordance of regional brain activity were found in both the treatment and control groups after EA. When performed at ST-36, EA produced activations in the middle prefrontal cortex, anterior cingulate cortex, postcentral gyrus, insula, lentiform nucleus, and cerebellum of healthy subjects [39]. In rats, increases in functional connectivity were found in the left striatum with the bilateral sensory, motor, auditory, visual cortex, hippocampus and left parietal, cingulate, retrosplenial cortex, and superior colliculus, as well as the right cerebellum after EA treatment at ST-36 [40]. Previous human studies have demonstrated that PE patients had increased brain activity in the attentional network and higher activation in the right middle temporal gyrus, as well as higher functional connectivity in the bilateral middle cingulate cortex, right middle frontal gyrus, and supplementary motor area [41, 42]. Patients with PE exhibited increased fALFF in the right inferior frontal gyrus (opercular part) and middle frontal gyrus, and these changes were associated with the level of disease severity, which suggested that increased brain activity might play a critical role in the development of PE [42]. In addition, PE patients with high sympathetic activity showed increased ReHo in the default mode network and auditory network, as well as enhanced ReHo in the right middle fontal gyrus, which suggested that increased neuronal activity might be associated with the high sympathetic activity of PE patients [43, 44]. Abnormal ALFF and ReHo values of PE patients recovered after dapoxetine administration [45, 46]. In this study, the ejaculatory frequency of rats was reduced after treatment with EA at ST-36 for 4 weeks. We speculated that these changes might be achieved by inhibiting activity in brain regions involved in the process of ejaculatory behavior.

Although ST-36 is not typically a primary acupoint for treating PE, our animal studies have shown that EA at ST-36 significantly prolongs ejaculation time in PE-treated rats. This effect is likely due to several factors. First, ST-36 itself has demonstrated efficacy in treating PE by modulating neurotransmitter levels and autonomic nervous function, supporting its therapeutic potential for sexual dysfunction [14, 47]. Second, our study utilized a 4-week acupuncture regimen, longer than the typical 2-week treatment in other studies, which might enhance therapeutic effects through sustained physiological adaptations. Third, PE is closely associated with sympathetic nervous system activity [48]. Performing EA at ST-36 has been shown to inhibit sympathetic nervous function, which is crucial for managing PE [49, 50]. This finding is supported by observed changes in plasma NE levels, indicating significant impact on the autonomic nervous system [47, 51]. Finally, focusing on a single acupoint in animal studies helps isolate and increase understanding of specific mechanisms without interference from other points. Future research will explore multi-acupoint strategies to further elucidate the relationship between acupuncture and PE in both animal models and clinical trials.

Interestingly, after 4 weeks of treatment, rats in the PE group showed significantly reduced ejaculation frequency and notably prolonged ejaculation latency, while these parameters remained stable in the control group. We attribute these findings to several factors: first, acupuncture exerts bidirectional modulation on autonomic nervous function, enabling both inhibition and excitation. Second, acupuncture and moxibustion therapies are known to regulate meridian flow, harmonize Yin and Yang, and rebalance bodily energies. When Yin and Yang are in equilibrium, acupuncture serves primarily as a preventive health measure, preempting imbalances. Third, acupuncture enhances immune function, bolstering the body’s resilience to external stressors. Although both groups exhibited changes in brain regions before and after acupuncture, additional mechanisms may influence the normal group. This finding is likely closely related to the holistic nature of acupuncture treatment. These insights offer novel avenues for future investigation.

However, there were several limitations in this study. First, one potential limitation of our study was the lack of a sham EA group and positive drugs. This was also a major limitation in acupuncture studies in the past. The curative effect of acupuncture might be underestimated due to some curative effects of sham acupuncture used in the control group. Therefore, whether sham acupuncture should be used as a real placebo is controversial. Second, some other limitations existed, such as the relatively small sample size and short follow-up time. Due to the short lifespan of animals and the extended treatment duration, long-term follow-up studies were not conducted. However, we are currently conducting a related clinical study (No. NCT06172855) with plans to incorporate long-term follow-up assessments. This study represents a crucial component of our ongoing research endeavors. Third in the present study it was unclear whether the levels of the brain neurotransmitters involved in ejaculation could be modulated by EA; therefore, this question will be further explored in our subsequent studies. Finally, considering the small sample size, the results of this study were not subjected to multiple comparison correction.

## Conclusion

In conclusion, PE rats showed altered magnitude and homogeneity of regional brain activity, which might be associated with the development of PE. The condition of PE was demonstrated to be improved and abnormal brain activity was regulated by the treatment of EA at ST-36 in rats. These therapeutic effects might be achieved by EA inhibiting the activity in brain regions involved in ejaculatory behavior. These findings may enhance understanding of PE and contribute to new, targeted therapies for PE.
